# Experiences and challenges with the new European Clinical Trials Regulation

**DOI:** 10.1186/s13063-023-07869-x

**Published:** 2024-01-02

**Authors:** Thale D. J. H. Patrick-Brown, Josephine Bourner, Sabrina Kali, Marius Trøseid, Yazdan Yazdanpanah, Piero Olliaro, Inge Christoffer Olsen

**Affiliations:** 1 Clinic for Surgery, Inflammatory Medicine and Transplantation (KIT Research), Oslo University Hospital, Oslo, Norway; 2 Pandemic Sciences Institute, University of Oxford, Oxford, UK; 3 ANRS | Emerging Infectious Diseases, Paris, France; 4 Department of Research Support for Clinical Trials, Oslo University Hospital, Oslo, Norway

**Keywords:** Clinical trial, Government regulation, Pandemics, Epidemics, Multicenter trials

## Abstract

**Background:**

The new European Medicines Agency (EMA) Clinical Trials Information System (CTIS), based on the Clinical Trials Regulation (CTR EU 536/2014), came into full effect on 31 January 2022 and was intended to provide an easier, more streamlined approach to the registration of clinical trials taking place in Europe. Using the experience gained on the new regulatory framework from three multi-national European clinical research studies of outbreak-prone infectious diseases, this article describes the advantages and shortcomings of the new clinical trial submission procedure.

**Methods:**

We report the time to approval, size of the application dossier, and number of requests for information (RFIs) for each study. We also explore the experience of each study within the regulatory framework and its use of CTIS to document the real-world, practical consequences of the system on individual studies. The study assesses the experience of three multi-country studies conducted in Europe working within the EU and non-EU regulatory environments.

**Results:**

While the time to regulatory and ethical approval has improved since the implementation of the new regulation, the timelines for approvals are still unacceptably slow, particularly for studies being conducted in the context of an evolving outbreak. Within the new regulatory approval procedure, there is evidence of conflicting application requirements, increased document burden, barriers to submitting important modifications, and debilitating technical hurdles.

**Conclusions:**

CTIS promised to lower the administrative bar, but unfortunately this has not been achieved. There are challenges that need to be urgently confronted and addressed for international research collaborators to effectively manage health crises in the future. While the value of multi-national outbreak research is clear, the limitations and delays imposed by the system, which raise challenging ethical questions about the regulation, are prejudicial to all clinical research, especially publicly funded academic studies. This report is relevant to both regulators and clinical researchers. It is hoped that these findings can help improve pan-European clinical trials, especially for the purpose of epidemic preparedness and response.

**Trial registration:**

This paper references experiences gained during management of three pan-European trials: EU-SolidAct’s Bari-SolidAct (CT No. 2022-500385-99-00 - 15 March 2022) and AXL-SolidAct (CT No. 2022-500363-12-00 - 19 April 2022), and MOSAIC (CT No. 2022-501132-42-00 - 22 June 2022).

## Background

The new European Medicines Agency (EMA) Clinical Trials Information System (CTIS), based on the Clinical Trials Regulation (CTR EU 536/2014), came into full effect on 31 January 2022. From its earliest inception, the new system was not just meant to align trial documentation with the upcoming new legislation, but also to create an easier, more streamlined approach to the registration of clinical trials taking place (predominantly) in Europe. Since each nation is responsible for authorising trials that will operate in their own territory, it was hoped that the new system would prove simple to use and ease the burden of management by providing a single, centralised platform where regulators, investigators, and the public could freely access trial information, reducing the time required to address individual requests. The system was touted by the EMA as being a “single entry point for clinical trial application submission, authorisation and supervision in the EU” which would “facilitate the recruitment of trial participants by allowing sponsors and researchers to easily expand trials to other EEA countries” [1].

This article examines the experience gained through three study applications to illustrate advantages and shortcomings of the system, especially in the context of epidemic preparedness and response, and propose solutions to improve the system.

## Methods

The studies included are EU-SolidAct’s Bari-SolidAct (the first multi-national trial to transfer from the old Voluntary Harmonisation Procedure (VHP)/Clinical Trials Directive (CTD) to the new CTIS/CTR), and then AXL-SolidAct and MOSAIC, two of the first multi-national trials to gain approval as new applications under the CTR (Table 1).

**Table 1:** Descriptions of the three studies included in this paper

Study	Description
EU-SolidAct / Bari-SolidAct (EU CTR : 2022-500385-99-00)	• EU-SolidAct denotes the platform trial and is part of the EU-RESPONSE project (EU Horizon 2020 Grant no. 101015736) • Bari-SolidAct is a phase 3 sub-investigation looking into the efficacy and safety of the JAK-inhibitor baricitinib in hospitalised COVID-19 patients ◦ Submitted and approved under the CTD (CTD 2001/20/EC) through VHP in 14 European countries in the spring of 2021 [2, 3] ◦ 14 initial countries: Austria, Belgium, Czechia, France, Germany, Greece, Hungary, Ireland, Italy, Luxembourg, Norway, Portugal, Slovakia, and Spain ◦ Fully academic (Sponsor, Oslo University Hospital) ◦ Platform and trial arm were transferred from VHP governed by CTD to CTIS governed by CTR 15th March 2022
AXL-SolidAct (EU CTR: 2022-500363-12-00)	• Phase 2b trial assessing the efficacy of the AXL-inhibitor bemcentinib on hospitalised patients with moderate pulmonary COVID-19 • New arm in the EU-SolidAct platform trial • Submitted 19 April 2022
MOSAIC (EU CTR: 2022-501132-42-00)	• Observational cohort study of clinical and virological outcomes in human mpox virus disease (formerly monkeypox) • Sponsor: University of Oxford; Sponsor’s representative in Europe: French National Agency for Research on AIDS (ANRS)/Inserm; Sponsor’s representative in Switzerland: Geneva University Hospital • Launched in response to a request from the EMA Clinical Trials Coordination Group (CTCG) for an observational study to characterise the evolving outbreak across Europe • Collects only clinical data and research samples • Study aims to describe the clinical and virological outcomes of patients with laboratory confirmed mpox • Inside the EU: Classified as a Low Intervention Clinical Trial as data is collected on mpox patients who receive tecovirimat (a drug newly authorised under “exceptional circumstances” without being studies in an mpox population) • Outside the EU (UK and Switzerland, and France prior to the CTIS application): received approval as an observational trial • Study initially submitted in June 2022 in eight EU/EEA countries: Belgium, France, Ireland, Italy, Netherlands, Norway, Portugal, and Spain

We present the time (days) from application submission to approval per country for each study, and the median and interquartile range (IQR) for the time to approval in each study.

We describe the size and scope of the application dossier for EU-SolidAct’s Bari-SolidAct under VHP and all three studies under CTIS, including conflicting country-specific requirements.

We summarise the technical hurdles encountered in CTIS across the three studies, as well as the challenges encountered in the system to respond to requests for information (RFIs) and submit modifications, both in Phase I, consisting of general trial information, including protocol and drug/equipment information, and Phase II where individual member state specific information such as informed consent forms, insurance, recruitment arrangements, suitability of investigator and hospital are included. ‘RFIs’ refers to any questions raised by the member states and/ or ethics committees during either of these phases.

We also describe the impact of the CTR’s “Low Intervention Clinical Trial” classification on MOSAIC and the challenges of the system for platform trials.

## Results

### Approval timelines under VHP

The Bari-SolidAct VHP application took 56 days from submission to second phase approval and then required further approval in phase 3 at state level by national competent authorities (NCAs) and the ethics review committees (ERCs) in the 14 participating countries. Overall, the time from initial submission to phase 3 approval was a median of 158 days (IQR 105–193). Table 2 presents details by country.

**Table 2:** Days to approval for countries under the VHP system and transition from VHP to CTIS (Bari-SolidAct), and for an initial application under CTIS (AXL-SolidAct and MOSAIC)

Country	Bari-SolidAct VHP	Bari-SolidAct Transition	AXL-SolidAct Initial CTIS	MOSAIC Initial CTIS
Austria	253	71	-	-
Belgium	168	48	77	41
Czechia	182	51	84	-
France	83	43	78	41
Germany	211	48	-	-
Greece	92	43	79	-
Hungary	120	48	-	-
Ireland	174	51	98	86
Italy	110	48	-	16
Luxembourg	217	56	80	-
The Netherlands	-	-	-	56
Norway	91	43	80	82
Portugal	184	43	-	51
Slovakia	141	44	83	-
Spain	148	44	79	42

The central VHP application consisted of 9 documents: cover letter, protocols, EudraCT form, summary of product characteristics (SmPC), investigator’s brochure (IB), good manufacturing practice (GMP) documents, and investigational medicinal product dossier (IMPD). Local documents were similar to those required in CTIS including translated label, patient information and informed consent forms, insurance, VHP approval letter, cover letter, curriculum vitae of the national coordinating investigator, and other country-specific documents according to local requirements. The total number of submitted documents was well below 200.

### Transferral of the EU-SolidAct/Bari-SolidAct trial from VHP to CTIS

The transfer submission of the Bari-SolidAct trial followed instructions issued by the European Commission in the “Clinical Trials Regulation (EU) No 536/2014 Questions and Answers” (CTR Q&A of April 2022) Question 11.8 answer 466 and 468 [4]. Therefore, we submitted the following documents in Part I:

- Cover letter
- Consolidated protocols (master and baricitinib-specific)
- IB for baricitinib (two versions)
- GMP relevant documents
- IMPD-Q

For Part II, only the latest approved versions of the subjects’ information sheets and ICFs for each country were submitted, per requirement for transferral of a trial from VHP to CTIS. The total transfer dossier consisted of 84 documents. The assessment, including Part I and Part II, which run concurrently to one another, took a median of 48 (IQR 43 to 51) days to decision. Days to conclusion were calculated by counting the number of days between the date of submission (both Parts I and II are submitted together) and the date of final approval of Part II in the individual countries involved as reported in the CTIS system. Part I approval runs concurrently to Part II approval but is only approved by the Reporting Member State. See details by country in Table 2.

Around the time of the transferral submission, the Bari-SolidAct trial had to stop the inclusion of immunocompetent patients due to external evidence from the RECOVERY trial showing lack of efficacy for baricitinib in this population [5]. This meant a substantial protocol amendment, triggering the requirement for a complete dossier to be submitted per the CTR Q&A. The full dossier consisted of nearly 800 documents, almost 10 times the size of the initial transfer dossier.

### New high-intervention trial application—AXL-SolidAct

For comparison, the AXL-SolidAct arm (EU CT no. 2022-500363-12-00) was submitted in 10 countries: Norway, Slovakia, Belgium, Czechia, France, Greece, Ireland, Italy, Luxembourg, and Spain. The median time to decision was 80 days (IQR 79–84). Note that the schedule for this submission also followed the CT-CURE timelines which sped up the time to approval significantly (see Table 2). The application dossier consisted of 535 documents for 10 countries and 54 sites.

### New Low-Intervention Clinical Trial application—MOSAIC

The first week of May 2022 is accepted as the beginning of the mpox outbreak in typically non-endemic countries [6]; the EMA requested organisation of a multi-country observational study on 20th May. A protocol was rapidly developed, and the MOSAIC trial was submitted as a Low-Intervention Clinical Trial (LICT) application in CTIS on the 22nd of June 2022 when mpox cases were on a steep rise.

Before the CTIS application could be submitted, a study team of five individuals from the University of Oxford and ANRS were required to undertake a 2-week extensive training course concurrent to compiling the necessary Part II documentation which added to the pressure involved in preparing an urgent application. The training was required to be completed prior to sending uploading the dossier which eventually contained 329 documents for 8 countries.

Once the initial application was submitted, the median time to decision was 46.5 days (IQR 41 to 62; see Table 2). In comparison, it took 14 and 20 days to get approval in UK and Switzerland respectively where it was considered an observational study, and 13 days in France, where it was submitted as an observational study in parallel with the CTIS application.

### Conflicting application requirements

The CTIS Sponsor handbook describes CTIS as a “harmonised and simplified” clinical trial application system [7]. However, only the initial uploading of documents is “harmonised”. Following this, individual MS requirements are fragmented and devolved, often generating a lack of consistency on requirements, especially during the request for information (RFI) phase (see Table 3).

**Table 3:** National CTIS requirements and related issues

Country	Requirement	Issue
Ireland	Searchable documents without signatures (not scanned documents)	Opposite from the requirement in Italy
	Submission of a Data Protection Impact Assessment (DPIA)	Not a pre-defined document, nor has it been requested by other participating countries
Italy	Scanned documents with (ink) signatures	Opposite from the requirement in Ireland
Hungary	Submitted protocol to be signed by all Hungarian investigators.	The submitted version of the protocol will usually be amended during the assessment phase, triggering versioning issues.
Luxembourg	A copy of the eCRF	Operationally it is more efficient to start the eCRF work after submission. To require a version compatible with the approved protocol while said protocol is subject to change induces versioning issues.
Luxembourg and Greece	Signed versions of the clinical trial agreements between the sites and the sponsor	This usually requires the agreement to be fully signed and executed. Many countries will not sign a contract before the application is fully approved, which creates a vicious circle of those who do not want to sign a contract before approval and those demanding signed contracts in CTIS.
Czechia	The full national dossier must be submitted for the transferal application	A clear violation to the requirements set forth in section 11 of the CTR Q&A document.
Spain	A unique, local version of the Site Suitability document	Will not accept the template developed by the EU Clinical Trials Expert Group endorsed by the EU commission.

### Issues with requests for information (RFIs)

Deadlines for RFIs vary. The median time between an RFI being raised and its deadline for response in MOSAIC was 12 days; however, on two occasions RFI deadlines for MOSAIC were unreasonably short (Portugal, 2 days; Belgium, 1 day). Given that each RFI can require changes to a large number of documents, the risk of lapsed RFIs is high.

### Technical hurdles

Numerous technical problems have been encountered within CTIS, many of which were major issues that should have been caught prior to roll-out; some of these are described in Table 4.

**Table 4:** Selected technical issues

Issue	Description
Non-substantial modifications	It is not possible to submit non-substantial modifications to the protocol, in contrast to the information given in the CTR Q&A, Annex IV. Currently, a substantial modification must be used for this purpose, which is time- and resource-intense.
Public website inconsistencies	The public website does not provide the latest approved versions of the documents. AXL-SolidAct, protocol version 1.4 was approved after submitting an SM but when downloading the full trial dossier from the public site an older version 1.3 is included. The same applies to the ICF. This is a major issue because stakeholders (i.e. patients) will access incorrect versions of essential trial documents. One formal document presenting the up-to-date situation with the latest approved version of documents and listing which countries are approved (and on which date) would be helpful as many peripheral entities require this information, such as data protection officers, pharmacies, and drug handling partners.
Part 1-Only Approvals	The technical solution to submitting a SM while there are Part I-only member states included is not yet implemented (as promised by the CTR Q&A document Question 3.6). When the first SM for AXL-SolidAct was submitted, four previously approved countries (Germany, Austria, Hungary, and Portugal) had to be withdrawn. Adding these countries later will cost a further 4 months.
Workarounds	In the MOSAIC study, it was not possible to update the Part II dossier in response to an RFI due to a technical fault in the system that lasted several weeks. On the advice of the CTIS management team, and to avoid a lapse in the RFI, 167 updated study documents had to be appended to the RFI itself, with a promise that they would be uploaded when the issue was fixed.
CTIS not a primary registry	CTIS is not registered as a primary trial registry in the International Clinical Trials Registry Platform (WHO ICTRP), and therefore do not fulfil the formal requirements scientific journals have for preregistration of clinical trials. Under CTD the EudraCT registry was a primary registry.

## Discussion

The EU-SolidAct platform trial with the Bari-SolidAct and AXL-SolidAct sub-investigations was the first trial to transfer from the VHP to the CTR (Bari-SolidAct), and one of the first multi-national trials to submit a new application to CTIS (AXL-SolidAct). The MOSAIC study was the first LICT to be submitted in the context of a multi-country outbreak and Public Health Emergency of International Concern (PHEIC). Thus, our experiences provide a unique insight into the deficiencies of the new regulation and its submission system (CTIS) during an epidemic.

### The application process

While the time from submission to approval is seemingly shorter with CTR/CTIS than with CTD/VHP, which was one of the objectives of overhauling the system [8], review timelines are still unacceptably slow especially for research that takes place within the context of an evolving outbreak. Individual reporting member states (RMSs) have tried to advocate for consistency and urgency from participating countries, for example in Bari- and AXL-SolidAct, the RMS (Norway) attempted to hold all participating countries to the COVID-19 CT-CURE initiative’s timelines. However, not all participating countries were part of CT-CURE, which meant there was an inconsistent approach to the review timelines leading to inevitable delays. This raises concerns about the preparedness of the system to respond to even more pressing situations.

In the early phase of the COVID-19 pandemic the Nor-Solidarity trial [9] took 3 days from submission to regulatory and ethical approval by the Norwegian authorities. Even before the mpox outbreak had been declared a PHEIC, the British and Swiss authorities had approved the MOSAIC study. While Italy showed that a relatively swift approval is possible (see Table 2), the general approval procedure in CTIS has taken so long that approvals for several countries in both trials were only received once their respective outbreaks were tailing off, precluding prospective enrolment, and preventing important modifications to the protocol that could have, for example, shed light on mpox transmission and immune responses in Clade IIb.

At the same time, the burden on applicants has increased. The number of documents required for a submission has markedly increased mainly for Part II (national level). Examples of new documents previously not required in many countries are individual site feasibility forms, CVs, declarations of interest for all investigators, recruitment arrangements, financial arrangements, data protection statements, and description of use of biological samples. Note that none of these documents are required in the UK and Switzerland. While some of these documents may be gathered as part of the trial file, they are not typically submitted for approval. Their inclusion in the submission dossier increases the number of documents that must be curated, reviewed, and potentially queried, increasing preparation and review timelines. This is not feasible in an outbreak context and is unnecessary.

### Platform trials and their associated legal snags

When planning for the Bari-SolidAct submission, the study team discussed how the submission of a platform trial with several arms should be managed with regulators and the EMA. Following advice from the EMA, the new arm was submitted as a separate trial, AXL-SolidAct, referring back to the master protocol as submitted in the original Bari-SolidAct trial. This advice has later been formalised in the “Complex clinical trials – Questions and answers” version 2022-05-23 (EMA/298712/2022). While this offers flexibility, it also introduces some legal issues; many of the centres that were already included in the platform were required to begin contracting de novo because the CTIS system has a “one application number, one contract” policy, where an amendment would have been preferable since the conditions of participation had not changed. Further, when the original Bari-SolidAct trial ends, changes to the protocol will no longer be possible; the master protocol will need to be transferred to the active AXL-SolidAct arm, which will presumably require a substantial modification (SM), and any other arms that may exist at that time will also need SMs to bring them in line, creating a logistical nightmare for the sponsor.

### The modification process

One major systematic hurdle for study teams is managing modifications, in particular smaller modifications such as adding new sites or changing PIs. Modifications of any nature cannot be submitted until both Parts I and II have been approved by the RMS and each member state (MS), respectively. This process prevented MOSAIC from implementing an important amendment to the protocol—specifically, a change needed to understand viral transmission and immune responses of mpox. For several weeks, the study team could only watch as case numbers in the outbreak dropped and the opportunity to collect important data faded. It is now unlikely these important research questions will be answered.

Adding new sites also poses challenges. New sites must be added using a SM and be approved by both regulators and ethics boards before the site can start enrolling patients (CTR Chapter III, Article 15). In other non-EU countries, such as the UK, this type of amendment is, in most cases, considered “non-substantial” and receives automatic approval. AXL-SolidAct has been able to recruit 8–10 new sites and MOSAIC has recruited 29 new sites, but these sites have been prevented from including patients for several months because existing amendments/approvals are caught in the pipeline. This has led to a critical loss of data that would have made a meaningful contribution to the objectives of both studies.

Where modifications are made, each SM can take 60–80 days, which seriously delays important inclusions and impedes necessary changes. This risk is particularly salient in the case of an epidemic, limiting the ability of a trial to evolve and adapt to an outbreak; large numbers of patients who could potentially contribute important data or benefit from new treatments slip through our fingers while changes are processed.

### Technical challenges

There are many obvious technical problems with CTIS, as undoubtably many users of the system have experienced. It is important to highlight that some of these technical issues have led to delays in study approvals, unnecessary raising of RFIs, and duplication of substantial amounts of work. While consideration of RFIs is an important part of the process governing the approval of a study, rules governing the RFI process are disproportionately punitive. When investigators fail to meet a deadline for response to an RFI, they are met with severe consequences. For example, AXL-SolidAct failed to meet the deadline for an early RFI during Part II assessment concerning a small change to an insurance document in Italy. As feedback was not received from the insurance vendor in time, the RFI lapsed, and consequently Italy was entirely removed from the application. This meant the Italian dossier had to be resubmitted from the start again despite having already received Part I approval. As sponsor, we take full responsibility for this delay, but the consequence of this small lapse was that Italy’s approval was delayed a further 4 months.

Further, when inconsistencies in the paperwork required are identified by ethics committees or regulators via RFI, there is no way of contacting the responsible ethics committees to request clarifications, which are often necessary as the requests are usually explained in just one or two sentences. The MOSAIC study was able to make much swifter progress with those ethics committees with whom the study team had direct contact than those who were anonymous and uncontactable.

Beyond those issues listed in Table 4, many other technical issues exist, not the least of which is the absence of appropriate support for platform trials, where amendments in different intervention arms block each other. Since platform trials are increasingly used in epidemic research, this problem needs immediate attention.

### Transparency

A welcome feature of CTIS is making, in principle, all submitted documents publicly available, along with its high threshold for deferrals. While we also adhered to this principle before the transition by publishing protocols, ICFs, and many other essential trial documents on our webpages, having a single hub where the whole regulatory and ethical dossier of a trial is available makes a huge difference. However because of this potential, it is very damaging that the public webpage is broken and currently showing a mix of current and superseded documents. While the technical issues behind the problems will hopefully be solved, it points to the basic immaturity of CTIS that such errors were not noted during the many years of preparation and have not been fixed in the 11 months since release.

### A proposal for change

In addition to the increased document burden, we have reported several other systematic hurdles. Some are embedded in the CTR and should be dealt with in the longer term, while others can and should be corrected quickly. Below are proposals for key changes that will ease the burden on participants in trials within Europe.

#### RFIs

It should be possible to re-open a lapsed RFI. At the very least, a Part II lapse should not force a country to begin from Part I approval yet again, which wastes precious time during an outbreak.

#### Harmonisation

National requirements should be further harmonised, or alternatively, the EMA could provide one central document stating all the country-specific document requirements, including payment information. Currently, it is very difficult for the sponsor of a multi-national trial, and even the Clinical Trials Units within MSs, to identify all national requirements before receiving an RFI, which then causes stress as sites are pressed to produce previously unknown documents within tight RFI deadlines. The fact that ethics committees are choosing to resolve queries directly with sponsor organisations outside of CTIS is an indicator that an impersonal approach to the complex topic of a clinical trial application does not work.

#### Amendments

It should be possible to make substantial and non-substantial modifications to a trial after approval of Part I, including changes to the protocol and other study documentation, and to add new countries without withdrawing previously approved countries. Given the inflexibility of the current system, we suggest that rather than being the responsibility of the submission system, coordination of Part I and II dossiers should be the responsibility of the sponsor. This would allow research to be implemented and adapted according to the needs of the study—not the limitations of CTIS.

#### Pricing of CTIS changes

Both initial applications and subsequent modifications are expensive, and very little actual information is available on websites. For example, a recent amendment, which was a minor change, cost €1750 to submit in Slovakia. Since SMs are now required to make even the smallest change, this becomes a significant and unpredictable financial burden on the Sponsor. Academic sponsors with limited budgets may especially find themselves in a difficult situation if they have to submit numerous substantial amendments each year to keep up with changing information, or with the evolving nature of an epidemic.

#### Equality in participation

The complexity and cost of procedures is especially prejudicial to publicly funded, independent research addressing important clinical and public health priorities, compared to pharmaceutical company-sponsored studies that can mobilise the required human and financial resources. This seriously jeopardises independent research, and potentially excludes less well-funded sites/countries. We feel strongly that the EMA could limit inequality by taking a more proportionate approach to minor trial updates.

#### LICT status

Finally, there should be a more proportionate approach taken to the requirements for a LICT in the CTR. In MOSAIC, there is no intervention being trialled and yet it must comply with the CTR in the same way as a trial evaluating an IMP. Moreover, the current LICT definition, which exists in no other jurisdiction, creates considerable complications for multi-national studies, impeding their conduct. An urgent reassessment of the definition of a LICT and its associated regulatory responsibilities needs to be undertaken to facilitate research in the EU and allow collaborative research to take place with non-member states.

#### Limitations and generalisability

Finally, a note should be made about the limitations and generalisability of this study. As described, our findings are limited to three studies enacted during a public health emergency situation. It would be useful to collect information on experiences from other groups, both in similar and non-emergency settings, for example non-communicable diseases, where perhaps the sense of urgency might be different, and with different trial designs. The technical issues we have observed, or similar issues, will probably have been observed by other researchers as well. Currently, there are 123 open issues (68 for Sponsor workspaces, and 55 for Authority workspaces) listed in the “Lists of known issues and proposed workarounds” [10], last updated January 2023. Therefore, although we may have presented some specific situations that are not globally ubiquitous, we have reported on issues that affected three large studies, making this paper indicative of the current situation for multi-national studies.

## Conclusion

In this paper, we have presented the advantages of the CTR as implemented via CTIS in comparison to the old CTD/VHP system, but also its substantial limitations. While it is difficult to directly compare the different scenarios, our results indicate that while the time to regulatory and ethical approval has decreased, the timelines for approvals are still unacceptably long, particularly for studies conducted in the context of an evolving outbreak, where speed and flexibility are essential. Conflicting and often burdensome requirements are a significant problem for the efficient management of multi-national trials. Associated issues such as convoluted legal contracting, overflowing queues for critical SMs, and technical errors are adding complications to the already arduous and time-consuming process of running trials.

While we have made several practical suggestions for the improvement of the new system, these do not address the ethical challenges it imposes. The regulation and associated processes have limited the prospective data collection for all three trials and prevented important changes that would have improved their scientific value. The EMA should consider whether priority is to be given to the process of ensuring member states are all aligned before a change is initiated, or rather to urgent public health issues by allowing changes in approved countries while the others are still deciding. Through working together to examine and resolve the issues blocking timely approval of trials, Europe’s medical community as a whole will be better positioned to respond quickly to emerging health crises, giving a better chance of capturing critical data in fast-evolving situations such as those experienced in pandemic situations.

## Data Availability

The dataset(s) supporting the conclusions of this article is(are) available in the Open Science Framework (OSF) repository, permanent link: https://osf.io/twupx.

## Abbreviations

CTIS: Clinical Trials Information System
RFI: Request for information
SM: Substantial modification
NSM: Non-substantial modification
CTR: Clinical trials registration
CTD: Clinical trials directive
VHP: Voluntary harmonisation procedure
LICT: Low-intervention clinical trial
NCA: National competent authority
ERC: Ethics review committee
SmPC: Summary of Product Characteristics
GMP: Good Manufacturing Practice
IMPD: Investigational Medicinal Product Dossier
IM: Investigator Brochure
IQR: Interquartile range
EMA: European Medicines Agency
EEA: European Economic Area
EU: European Union
